# Predictors of mortality in adult people living with HIV on antiretroviral therapy in Nepal: A retrospective cohort study, 2004-2013

**DOI:** 10.1371/journal.pone.0215776

**Published:** 2019-04-23

**Authors:** Mirak Raj Angdembe, Anjana Rai, Kiran Bam, Satish Raj Pandey

**Affiliations:** Saath-Saath Project, Nepal, Kathmandu, Nepal; Gabriel Toure University Hospital, MALI

## Abstract

**Background:**

In Nepal, since 2004, 19,388 people living with HIV (PLHIV) have been enrolled on antiretroviral therapy (ART). The aim of this study was to measure mortality rate and to identify predictors of mortality in adult (≥15 years) PLHIV who initiated ART between 2004 and 2013 in five large ART centers of Nepal.

**Methods:**

This retrospective cohort study of 3,799 (60.5% male) adult PLHIV uses secondary data collected from standard ART registers. Time from ART initiation (baseline) to death or censoring (loss to follow-up or December 31, 2013) was assessed. Mortality rates per 100 person-years were calculated. Kaplan-Meier models were used to estimate the probability of mortality over time. Predictors of mortality were determined using Cox-regression models.

**Results:**

The overall mortality rate was 6.98 (95% CI: 6.46–7.54) per 100 person-years, 4.11 (95% CI: 3.53–4.79) in females and 9.14 (95% CI: 8.36–9.99) in males. Mortality rates were higher in early months after ART initiation, particularly in the first three months. Baseline predictors of mortality were ART center, male gender (adjusted HR = 2.08, 95% CI: 1.69–2.57), residence outside the ART district (AHR = 1.45, 95% CI:1.19–1.76), World Health Organization clinical stage III (AHR = 1.67, 95% CI: 1.13–2.46) and IV (AHR = 2.21, 95% CI: 1.45–3.36), bedridden <50% time in the last month (AHR = 1.92, 95% CI: 1.52–2.41), bedridden >50% time in the last month (AHR = 3.82, 95% CI: 2.95–4.94), lower bodyweight/kg (AHR = 1.04, 95% CI: 1.03–1.05), CD4 count <150 cell/mm^3^ (AHR = 2.14, 95% CI: 1.05–4.34) and treatment not switched to second-line regimen (AHR = 3.05, 95% CI: 1.35–6.90).

**Conclusions:**

Mortality rates were higher soon after ART initiation, particularly in males and gradually decreased over time. Poor baseline clinical characteristics were significantly associated with higher mortality. Increased ART coverage with decentralization of sites to lower levels including community dispensing, differentiated and improved service delivery and initiation of ART at a less advanced disease stage may reduce early mortality.

## Introduction

Globally, 17 out of 36.7 million people living with HIV (PLHIV) had access to antiretroviral therapy (ART) in 2015 [1]. With increased service coverage and sustained access to ART, new HIV transmission is being averted, preventing millions of AIDS related deaths worldwide. An estimated 7.8 million AIDS related deaths were averted between 2000 and 2014 due to ART roll out. This includes 5.2 million deaths in low and middle-income countries [2].

In 2016 in Nepal, the adult (15–49 years) HIV prevalence was estimated to be 0.17% in the general population, reduced from 0.35% in 2005 [3]. The epidemic is mainly concentrated among key populations: male and female sex workers (FSW) and their clients, people who inject drugs (PWID), male labor migrants (MLM) and their wives, men who have sex with men (MSM), transgender (TG) people and prison inmates. The Integrated Biological and Behavioral Surveillance (IBBS) surveys conducted in Nepal from 1999 to 2018 indicate that HIV prevalence among key populations has either stabilized or decreased considerably in most groups. Among FSW, HIV prevalence was 2.2% in Kathmandu valley (2017) [4], compared to less than one percent in Pokhara valley (0.3%) (2016) [5] and in 22 terai highway districts (0.7%) (2018) [6]. Among PWID, HIV prevalence was highest in Kathmandu valley (8.5%) (2017) [7] followed by the western terai (5.3%) (2017) [8] and Pokhara valley (4.9%) (2017) [9]. HIV prevalence was lowest among PWID in eastern terai (3.3%) (2017) [10]. Among MLM, HIV prevalence was less than one percent (0.4%) in the western and mid to far western regions (2017) [11] and in the eastern region (0.3%) (2018)[12]. Among wives of migrants HIV prevalence was 0.5% (2018) in the far western region[13]. HIV prevalence among MSM and TG people had remained stable in Kathmandu valley at around four percent or below between 2004–2012 but in recent years, has increased from 2.4% (2015)[14] to 6.2% (2017) [15]. In terai highway districts, HIV prevalence among MSM and TG people has remained stable at 8.2% in 2016 [16] and 2018 [17].

In 2004, ART services were initiated free of charge from Sukraraj Tropical and Infectious Disease Hospital in Kathmandu. Since then, there has been a rapid expansion of services and as of May 2018, there were 74 ART centers across the country [18]. Despite this increase in ART access, and although treatment coverage is on the rise—from 31.6% in 2016 to 44.4% of the estimated 32,735 PLHIV in 2017—the overall coverage remains low [3, 19, 20]. Yet, increased treatment uptake has contributed to a decrease in deaths due to AIDS-related illness. In 2016, an estimated 1,771 deaths were caused by AIDS, compared to 2,263 deaths in 2015 [3]. Of those ever enrolled in ART, 76% were alive and were still on treatment after 36 months of treatment [19].

In Nepal, since 2004, 19,388 PLHIV have been ever enrolled on ART (as of July 2017) [19]. Once on ART, with effective adherence, viral replication is suppressed leading to restoration and preservation of immune function. Over time, morbidity and mortality is reduced with improved quality of life. However, compared to developed countries, in resource poor settings, mortality rates are higher in the first months of ART [21–25]. Previous studies have reported several predictors of mortality among those on ART, including baseline levels of HIV RNA [26], WHO clinical stage at the start of treatment, body mass index, anemia, CD4 cell count [27–33], cotrimoxazole prophylaxis [34], viral load [33], sex [35, 36] and adherence [37, 38]. There may be regional differences [21] in these predictors as delivering sustained ART in resource poor settings is a challenging task. In particular, major constraints include access to health facilities, shortages of healthcare staff; inadequate availability of drugs; weak health systems and laboratory capacity; and poor health data management systems causing difficulty in monitoring the PLHIV [39–41].

Predictors of mortality are poorly understood in Nepal. In the Far Western region, higher mortality was reported within the first three months of ART initiation and among those with poor baseline clinical characteristics, particularly men [23]. However, the ART centers across Nepal may be different in terms of access and quality of services and there may be variation in background characteristics of the PLHIV attending these centers affecting outcomes for those on treatment. Because Nepal has started providing ART for all individuals with HIV, it is increasingly important to understand survival and program retention over longer follow-up periods and across different centers. This understanding can contribute to reduce mortality among those on ART. The aim of this retrospective cohort study was to measure mortality rate and to identify predictors of mortality in adult (≥15 years) PLHIV who initiated ART between 2004 and 2013 in five large ART centers of Nepal—one each from the five development regions of the country.

## Materials and methods

### Ethics statement

The study protocol was reviewed and approved by the Protection of Human Subjects Committee, institutional review board of FHI 360 in Durham, North Carolina, USA and Nepal Health Research Council, local ethical review board in Kathmandu, Nepal. Approval was also obtained from all five ART centers. Confidentiality and anonymity were maintained at all stages of data collection. All PLHIV records were fully anonymized. To ensure confidentiality, each PLHIV’s record was provided a unique identification number which was used only during data analysis. This number did not link with any other information about the PLHIV. The site registration number was used to track the records from one register to another. Identifying information including first name was not collected.

### Study design and sites

**Fig 1:**
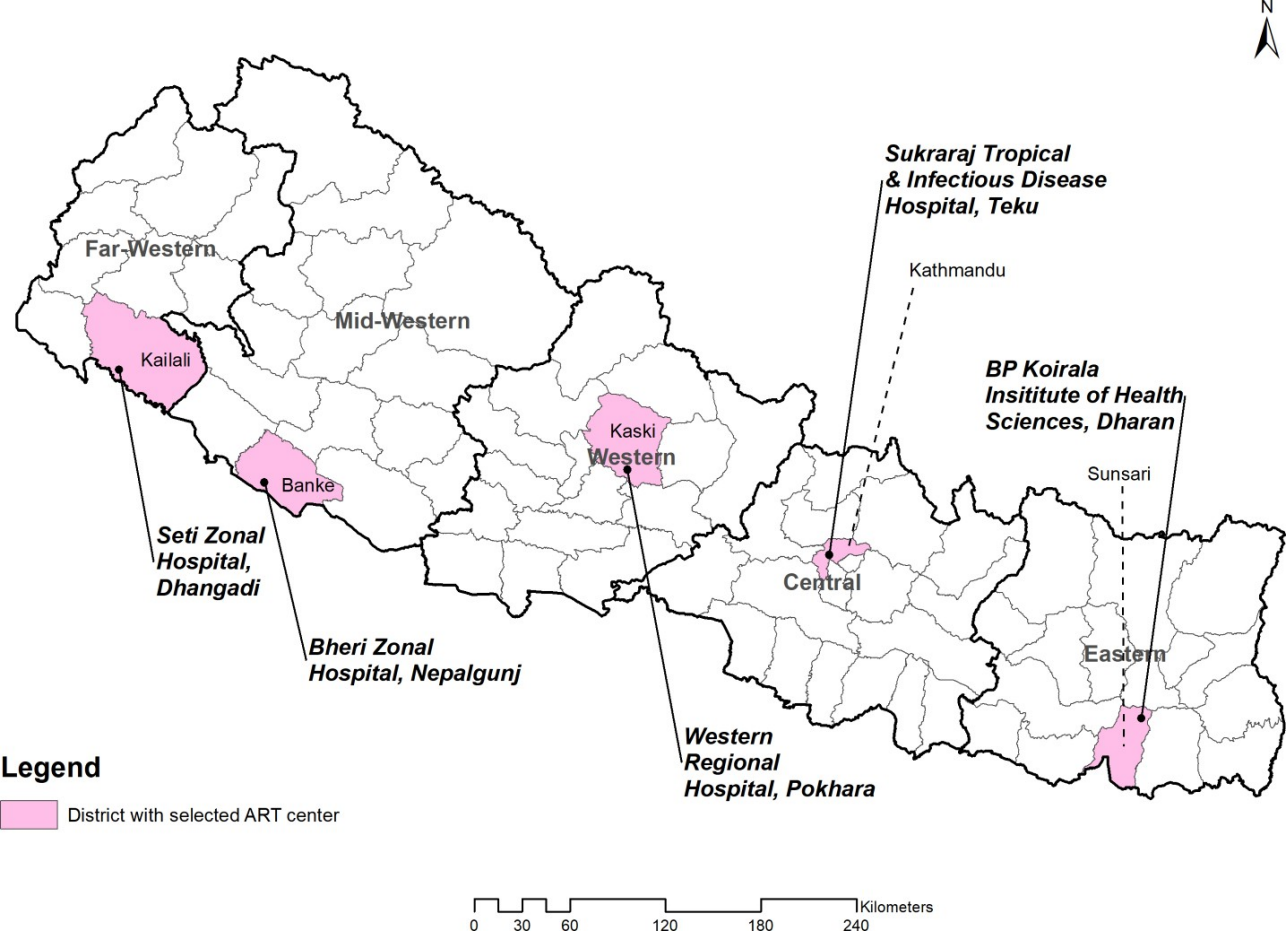
Sampled ART centers in each development region.

This was a retrospective cohort study. As of May 2018, there were 74 ART centers across Nepal’s five development regions: Eastern (12 centers), Central (20 centers), Western (17 centers), Mid-western (11 centers) and Far-western (14 centers). For this study, it was not feasible to include all ART centers, so one ART center was purposively selected from each development region: BP Koirala Institute of Health Sciences (BPKIHS), Sunsari from Eastern development region; Sukra Raj Tropical and Infectious Disease Hospital (STIDH), Kathmandu from Central region; Western Regional Hospital (WRH), Kaski from the Western region; Bheri Zonal Hospital (BZH), Banke from Mid-Western region; and Seti Zonal Hospital (SZH), Kailali from Far Western region (Fig 1). This sampling strategy provided geographic representation of the country. Selecting the ART center per region with the biggest number of ART clients gave us the largest sample possible from a sub-set of centers.

### Study population

The study population consisted of all adult (≥15 years) PLHIV who started ART between January 1, 2004 and December 31, 2013 at the five selected ART centers. Excluded from the analysis were those who: (i) transferred in from another ART center to the study site; (ii) transferred out to another ART center; (iii) had a history of previous antiretroviral (ARV) treatment (received at any time in the past from anywhere) during enrollment in the ART center; (iv) were children (aged 0–14 years); or (v) missed their last follow up visit. PLHIV who had transferred in or out were excluded because it was not possible to collect either their start or end-point data due to challenges surrounding their documentation (illegible handwriting, deteriorated records, missing information). It was difficult to track them from one ART center to another even though they may be within the same national ART system. Those with previous ARV treatment history at the time of enrollment were also excluded for similar reasons. In the current National HIV recording and reporting system, those who miss their follow-up visits for more than three months are counted as loss to follow-up and the date of the last registered follow-up visit is recorded as date of loss to follow-up. Those who have missed their last follow-up visit were excluded as their outcome on ART could not be determined.

### Study variables

#### a. Outcome variable

The survival status of those who started ART between January 1, 2004 and December 31, 2013 at any of the five ART centers selected for this study. The date of ART initiation, date of loss to follow up and date of death of those who died from all causes while on ART were collected. Those who were on treatment and not dead on December 31, 2013 were considered to be alive. The primary outcome for analysis was death. All those who died within the study period were put in one group and those who were still surviving or lost to follow up were put in another group.

#### b. Time variable

The time from the date of ART initiation to the date of death or censoring (loss to follow-up or December 31, 2013). It was calculated in days (then converted to months and years).

#### c. Predictor variables

Background characteristics and clinical characteristics at the start of ART as recorded in the registers.

*Background characteristics*: Place of ART center, age (in completed years), gender (including third gender), ethnicity (based on last name), usual place of residence/address (district), risk of HIV transmission, education (literacy status), employment status, habit of alcohol consumption, marital status and partner’s HIV status (+ve /-ve /unknown). Risk of HIV transmission included: commercial sex worker, other heterosexual route, MSM, PWID, blood transfusion, mother to child and unknown as mentioned and recorded in Patient HIV Care and ART record/follow-up form.

*Clinical characteristics*: were assessed at the time of ART initiation and included performance scale, bodyweight (kg), WHO clinical staging, CD4 count (cells/mm^3^), ART start regimen, tuberculosis (TB) treatment during ART and whether treatment was substituted within first line drugs or switched to second line regimen.

Performance scale consisted of A- normal activity, B- bedridden <50% of the day during the past month, and C- bedridden >50% of the day during the past month. The health personnel at the ART centers use WHO clinical staging guidelines to assess and record the clinical stage. It is based on the load of clinical symptoms and infection and consists of four groups (stage I, stage II, stage III, and stage IV). The health condition progressively worsens from stage I to IV. The ART start regimens were grouped into three groups: Group 1: AZT-3TC-NVP or AZT/ZDV-3TC-EFV, Group 2: TDF-3TC-EFV or TDF-3TC-NVP and Group 3: d4T-3TC-EFV or d4T-3TC-NVP. Ethnicity was classified based on ethnic codes as defined by the Health Management Information System of Government of Nepal, also used by other published studies [42].

### Data collection

Secondary data were collected from standard registers maintained at the ART centers. The data collection took place from December 2014 to January 2015. Three types of registers were used at the ART centers. The Pre-ART register is where all PLHIV visiting ART center but not yet eligible for ART are registered and once they become eligible for ART and start receiving treatment, they are transferred to the ART register. The third register is the Patient HIV Care and ART record/follow-up register where individual clinical characteristics are recorded. Once ART is started, the first follow-up visit is scheduled after two weeks and then at a monthly duration. The records are updated during each follow-up visit. Data collection tool was pretested at an ART center in Lalitpur district, not included in the study sample of selected sites. It was realized that height of adults is not measured and hence, the variable was dropped.

### Data analysis

**Fig 2:**
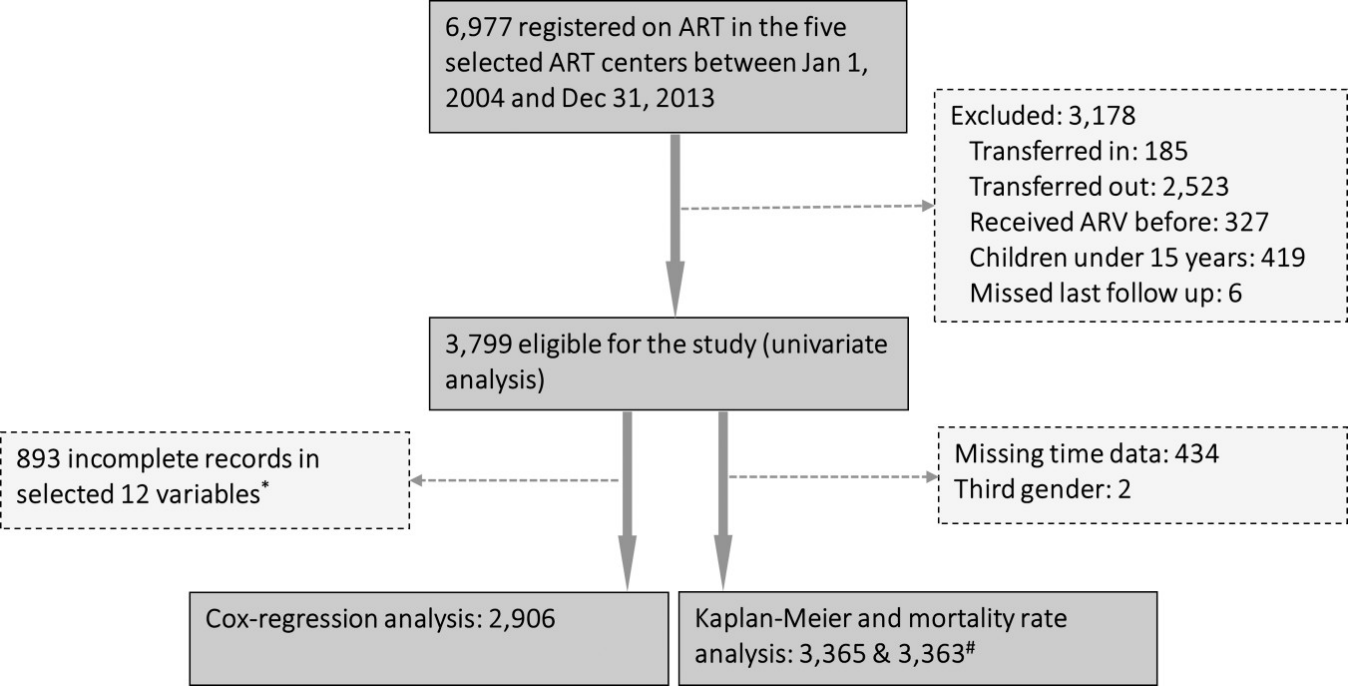
Profile of study cohort. * The 12 variables were (1) ART center; (2) Age; (3) Gender; (4) Ethnicity; (5) Usual place of residence; (6) Partner's HIV status; (7) WHO clinical staging; (8) Performance scale; (9) Body weight; (10) CD4 count at the start of ART; (11) TB treatment during ART and (12) Whether treatment was switched to second line regimen. ^#^Third gender excluded.

During the study period 6,977 PLHIV were registered on ART in the five selected ART centers. Of them 3,178 were excluded (185 transferred in, 2,523 transferred out, 327 had a history of previous ARV treatment, 419 children 0–14 years, 6 missed last follow up). Thus, 3,799 were eligible for the study. However, out of 3,799 cases, 434 had time data missing, even though the outcome (death) was known. Therefore, after excluding a further 434 people, a total of 3,365 cases were used for Kaplan-Meier analysis. However, for mortality rate analysis 3,363 cases were used–two third gender cases were also excluded because this number was too small for calculation of sex wise mortality rate. For the cox-regression analysis, 893 cases with incomplete records in 12 selected variables (ART center, Age, Gender, Ethnicity, Usual place of residence, Partner's HIV status, WHO clinical staging, Performance scale, Body weight, CD4 count at the start of ART, TB treatment during ART and whether treatment was switched to second line regimen) were excluded from the eligible population, resulting in 2,906 cases used (Fig 2).

Univariate analysis was carried out by examining frequency distribution (n/%) of the predictor and outcome variables. Mortality rates (per 100 person-years) were calculated. Kaplan-Meier (KM) curves were generated to examine the survival function after ART initiation. Hazard ratios (HRs) with 95% confidence intervals (CI) were estimated using Cox proportional hazards regression models. A *P*-value <0.05 was considered to be statistically significant. Data were analyzed using SPSS Version 17.0 and Stata SE Version 12.0. Mortality rates and KM curves were generated using Stata while rest of the analysis was performed using SPSS.

## Results

Between January 1, 2004 and December 31, 2013, there were 3,799 PLHIV on ART who were eligible for the study. Of these, 754 (19.8%) were lost to follow up, 2,294 (60.4%) were on treatment, three were stopped treatment and 748 (19.7%) had died. The causes of death were not documented. Thus, the results presented are of all-cause mortality.

### Background characteristics

At ART initiation, the 3,799 PLHIV on ART had a median age of 35 years (range, 15–80 years) and 41.1% of them were aged between 25–34 years. They were predominantly male (60.5%), currently married (73.8%), literate (57%), unemployed (78.1%), non-users of alcohol (89%) and belonged to ‘upper caste’ groups (42.6%). Many (41.3%) were from STIDH, Kathmandu and more than half (56.3%) resided in districts other than where the ART center was located. For most, the possible risk of HIV transmission was determined to be heterosexual (sex other than with commercial sex worker) (74.1%) and 64.6% of the sample reported their partner’s HIV status to be positive (Table 1).

**Table 1:** Summary of background characteristics of the cohort at ART initiation (Jan 2004-Dec 2013) (N = 3,799).

Characteristics	Total	Deaths
	n (%)^a^	n (%)^b^
**ART center**^**c**^		
BPKIHS, Sunsari	410 (10.8)	44 (10.7)
STIDH, Kathmandu	1571 (41.3)	281 (17.9)
WRH, Kaski	913 (24.0)	220 (24.1)
BZH, Banke	325 (8.6)	78 (24.0)
SZH, Kailali	580 (15.3)	125 (21.5)
**Age (years)** *(Median*, *range*: *35*, *15–80)*** **		
15–24	222 (5.8)	33 (14.9)
25–34	1562 (41.1)	274 (17.5)
35–44	1423 (37.5)	293 (20.6)
≥45	592 (15.6)	148 (25.0)
**Gender**		
Male	2299 (60.5)	553 (24.1)
Female	1498 (39.4)	193 (12.9)
Third gender	2 (0.05)	0
**Ethnicity (Last name)**		
Upper caste groups	1617 (42.6)	330 (20.4)
Relatively advantaged *janajati*	572 (15.1)	98 (17.1)
Disadvantaged *janajati*	795 (20.9)	145 (18.2)
*Dalit*	593 (15.6)	129 (21.7)
Disadvantaged non *dalit* terai caste groups	154 (4.0)	28 (18.2)
Religious minorities	68 (1.8)	18 (26.5)
**Usual place of residence (district)**		
Same as ART district	1661 (43.7)	309 (18.6)
Other than ART district	2138 (56.3)	439 (20.5)
**Marital Status**		
Unmarried/divorced/separated	678 (26.2)	143 (21.1)
Currently married	1912 (73.8)	329 (17.2)
*Not recorded*	*1209* (*31*.*8*)	
**Literate**		
Yes	1577 (57.0)	246 (15.6)
No	1190 (43.0)	239 (20.1)
*Not recorded*	*1032* (*27*.*2*)	
**Employed**		
Yes	584 (21.9)	63 (10.8)
No	2083 (78.1)	407 (19.6)
*Not recorded*	*1132* (*29*.*8*)	
**Alcohol use**		
Habitual	87 (4.1)	24 (27.6)
Social	146 (6.9)	30 (20.5)
No use	1890 (89.0)	306 (16.2)
*Not recorded*	*1676* (*44*.*10*)	
**Risk of HIV transmission**		
Commercial sex worker	164 (5.6)	30 (18.3)
Other heterosexual route	2153 (74.1)	382 (17.7)
MSM	19 (0.7)	4 (21.1)
PWID	327 (11.3)	51 (15.6)
Blood transfusion	56 (1.9)	6 (10.7)
Mother to child	15 (0.5)	2 (13.3)
Unknown	172 (5.9)	28 (16.3)
*Not recorded*	*893* (*23*.*5*)	
**Partner's HIV status**		
+ ve	761 (64.6)	134 (17.6)
- ve	417 (35.4)	86 (20.6)
*Unknown + Not recorded*	*2621* (*69*.*0*)	

^a^Column percentage excluding unrecorded cases

^b^Row percentage

^c^BPKIHS: BP Koirala Institute of Health Sciences; STIDH: Sukra Raj Tropical and Infectious Disease Hospital; WRH: Western Regional Hospital; BZH: Bheri Zonal Hospital; SZH: Seti Zonal Hospital; Not recorded percentage out of total 3,799 cases

The overall proportion of known death among those that had accessed ART was 19.7% (748 out of 3,799). Deaths were almost double (24.1%) among male compared to female (12.9%). Likewise, higher deaths were observed in BZH (24%) and WRH (24.1%), among ≥ 45 years’ age group (25%), religious minorities (26.5%), those residing in districts other than where the ART center was located (20.5%), unmarried or divorced or separated (21.1%), illiterate (20.1%), unemployed (19.6%), habitual users of alcohol (27.6%), risk of transmission MSM (21.1%) and among those with HIV negative partners (20.6%) (Table 1).

### Clinical characteristics

At the start of ART, around 13% were categorized under performance scale C (bedridden >50%). The median bodyweight was 49 kg (range, 21–98 kg) with nearly 12% weighing less than 40 kg. Around 41% and 16% were in WHO clinical stage III and IV respectively at the time of ART initiation. The median CD4 cell count was 136 cells/mm^3^ (interquartile range [IQR], 67–213 cells/mm^3^). Majority (78.9%) had started a Group 1 ART regimen i.e. either AZT-3TC-NVP or AZT/ZDV-3TC-EFV. For 26%, treatment was switched within first line drugs and around 2% had their treatment switched to second line regimen. Nearly 17% had received TB treatment during ART.

The proportion of death was higher among those having performance scale C (46.3%), bodyweight <40 kg (36.5%), WHO stage IV (41.3%), CD4 count <150 cells/mm^3^ (26.9%) and ART regimen—Group 3 (33.8%) at the start of ART. Similarly, deaths were higher among those who received TB treatment during ART (26.8%), those without treatment substitution within first line drugs (20.3%) and those that didn’t have their treatment switched to second line regimen (19.8%) (Table 2).

**Table 2:** Summary of clinical characteristics of the cohort at ART initiation (Jan 2004-Dec 2013) (N = 3,799).

Characteristics	Total	Deaths
	n (%)^a^	n (%)^b^
**Performance scale**		
A (Normal)	2008 (55.7)	222 (11.1)
B (Bedridden <50%)	1124 (31.2)	252 (22.4)
C (Bedridden >50%)	473 (13.1)	219 (46.3)
*Not recorded*	*194* (*5*.*1*)	
**Bodyweight (kg)** *(Median*, *range*: *49*, *21–98)*		
<40	422 (11.6)	154 (36.5)
40–49	1397 (38.4)	303 (21.7)
≥50	1817 (50.0)	236 (12.9)
*Not Recorded*	*163* (*4*.*3*)	
**WHO clinical stage**		
Stage I	508 (14.3)	38 (7.5)
Stage II	1020 (28.7)	89 (8.7)
Stage III	1449 (40.8)	342 (23.6)
Stage IV	574 (16.2)	237 (41.3)
*Not recorded*	*248* (*6*.*5*)	
**CD4 count (cells/mm**^**3**^**)** *(Median*, *interquartile range*: *136*, *67–213)*		
< 150	1956 (54.4)	527 (26.9)
150–199	581 (16.2)	68 (11.7)
200–349	948 (26.3)	70 (7.4)
≥ 350	111 (3.1)	10 (9.0)
*Not recorded*	*203* (*5*.*3*)	
**ART start regimen**		
Group 1 (AZT-3TC-NVP) (AZT/ZDV-3TC-EFV)	2979 (78.9)	494 (16.6)
Group 2 (TDF-3TC-EFV) (TDF-3TC-NVP)	195 (5.2)	57 (29.2)
Group 3 (d4T-3TC-EFV) (d4T-3TC-NVP)	402 (10.6)	136 (33.8)
Combination regimen not mentioned clearly	201 (5.3)	44 (21.9)
*Not recorded*	*22* (*0*.*6*)	
**Treatment substituted within 1**^**st**^ **line drugs **		
Yes	980 (25.8)	174 (17.8)
No	2818 (74.2)	573 (20.3)
*Not recorded*	*1*	
**Treatment switched to 2**^**nd**^ **line regimen**		
Yes	87 (2.3)	11 (12.6)
No	3712 (97.7)	737 (19.8)
**TB treatment during ART**		
Yes	637 (16.8)	171 (26.8)
No	3145 (83.2)	575 (18.3)
*Not recorded*	*17(0*.*4)*	

^a^Column percentage excluding unrecorded cases

^b^Row percentage; Not recorded percentage out of total 3,799 cases

### Mortality

Out of 3,799 PLHIV on ART, two were third gender and 434 had time data missing, even though the outcome (death) was known. These cases were excluded for calculation of sex specific mortality rates. Thus, of 3,363 PLHIV at risk 649 had died while receiving ART during 9,291 person-years of follow-up. The median years of follow-up after ART initiation was 2.3 years (IQR: 0.6–4.5 years).

The mortality rates for male, female and total PLHIV on ART are presented in Table 3. Over the study period, the total mortality rate was 6.98 per 100 person-years (95% CI: 6.46–7.54). In male cohort, the mortality rate was 9.14 per 100 person-years (95% CI: 8.36–9.99), while in the female cohort it was almost half (4.11 per 100 person-years [95% CI: 3.53–4.79]) over the study period. Highest mortality rate (44.64 per 100 person-years [95% CI: 40.15–49.63]) was observed in the first three months of follow-up since ART initiation. Mortality rates decreased as follow-up time intervals increased. Similar trends were observed in both male and female cohorts. However, the overall and follow-up time interval-based mortality rates were much higher for male compared to female.

**Table 3:** Mortality rates per 100 person-years over different follow-up time intervals (N = 3,363).

Follow up time intervals	Mortality rates per 100 person-years at risk (95% CI)		
	Male (n = 2011)	Female (n = 1352)	Total (n = 3363*)
0–3 months	58.11 (51.46–65.62)	25.74 (20.73–31.96)	44.64 (40.15–49.63)
0–6 months	38.74 (34.76–43.17)	15.55 (12.73–18.99)	28.94 (26.30–31.83)
0–1 years	24.23 (21.89–26.81)	10.06 (8.39–12.06)	18.15 (16.61–19.82)
0–2 years	15.69 (14.26–17.26)	7.21 (6.14–8.47)	12.02 (11.07–13.05)
0–5 years	9.81 (8.96–10.74)	4.46 (3.82–5.21)	7.51 (6.95–8.13)
0–8 years	9.14 (8.36–9.99)	4.10 (3.52–4.78)	6.98 (6.46–7.54)
**Over the study period**	**9.14 (8.36–9.99)**	**4.11 (3.53–4.79)**	**6.98 (6.46–7.54)**

* Two third gender cases were excluded from the analysis

Table 4 shows overall mortality rates in different ART centers including disaggregation by sex. Overall mortality rate was highest in BZH, Banke (11.46 per 100 person-years [95% CI: 9.11–14.42]) and lowest in BPKIHS, Sunsari (3.89 per 100 person-years [95% CI: 2.75–5.50]). Mortality rates in BZH, Banke and WRH, Kaski (9.20 per 100 person-years [95% CI: 7.97–10.63]) were higher compared to the total mortality rate (6.98 per 100 person-years). Mortality rates were higher for male compared to female in all five ART centers.

**Table 4:** Mortality rates per 100 person-years over the study period in different ART centers (N = 3,363).

ART center	Mortality rates per 100 person-years at risk (95% CI)		
	Male (n = 2011)	Female (n = 1352)	Total (n = 3363*)
BPKIHS, Sunsari	4.69 (3.17–6.94)	2.41 (1.15–5.06)	3.89 (2.75–5.50)
STIDH, Kathmandu	7.29 (6.32–8.41)	4.65 (3.71–5.82)	6.27 (5.55–7.07)
WRH, Kaski	11.58 (9.79–13.70)	5.86 (4.43–7.76)	9.20 (7.97–10.63)
BZH, Banke	19.55 (15.36–24.88)	2.34 (1.11–4.91)	11.46 (9.11–14.42)
SZH, Kailali	10.26 (8.11–12.96)	2.70 (1.82–3.99)	5.95 (4.87–7.27)

* Two third gender cases were excluded from the analysis

**Fig 3:**
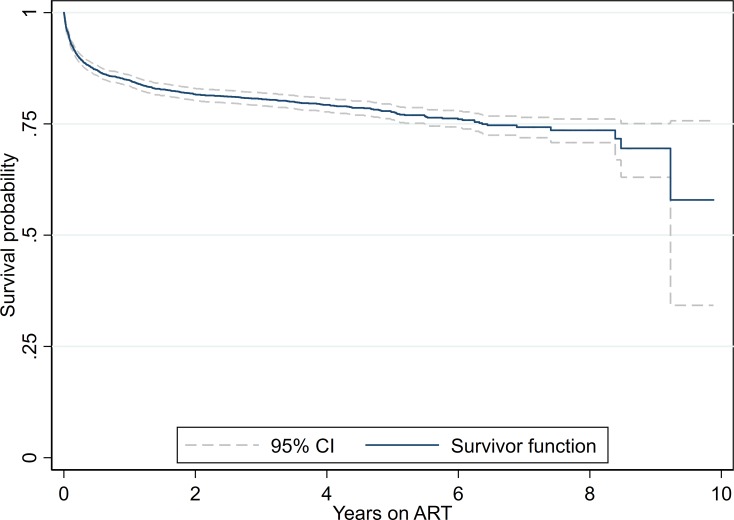
Kaplan-Meier survival curve of adult PLHIV on ART (N = 3,365).

Kaplan-Meier curve was fit to examine survival functions. KM-Survival curve of 3,365 adult PLHIV on ART is presented in Fig 3. It showed a decreasing trend of survival probability among adult PLHIV over follow-up time (time from ART initiation in years). The survival probability of PLHIV on ART at 3 months, 6 months, 1 year, 2 years, 5 years and at 8 years was 89.6% (95% CI: 88.6%- 90.6%), 87.1% (95% CI: 85.9%- 88.2%), 84.8% (95% CI: 83.5%- 86.0%), 81.7% (95% CI: 80.2%- 83.0%), 77.7% (95% CI: 75.9%- 79.3%) and 73.6% (95% CI: 70.8%-76.1%) respectively.

The relative hazards of mortality after ART initiation, both crude and adjusted, for several characteristics is presented in Table 5. In unadjusted analysis, mortality of adult PLHIV on ART was significantly associated with ART center, age, gender, ethnicity, usual place of residence, WHO clinical stage, performance scale, bodyweight, CD4 cell count, TB treatment during ART and treatment switch to second line regimen. Of them, age, ethnicity and TB treatment during ART did not remain significantly associated in adjusted analysis.

**Table 5:** Predictors of mortality in PLHIV on ART during 2004–2013 (N = 2,906).

Characteristics	n	Unadjusted		Adjusted	
		HR (95% CI)	*P*-Value	HR (95% CI)	*P-*Value
**ART center**^**a**^					
BPKIHS, Dharan	159	0.64 (0.38–1.07)	0.087	0.33 (0.19–0.56)	<0.001
STIDH, Kathmandu	1378	1.05 (0.82–1.36)	0.681	0.60 (0.44–0.82)	<0.001
WRH, Kaski	639	1.37 (1.04–1.79)	0.024	0.71 (0.51–0.97)	0.032
BZH, Banke	247	1.42 (1.00–2.01)	0.047	1.27 (0.87–1.86)	0.215
SZH, Kailali	483	1.00		1.00	
**Age (years)**					
15–24	162	1.00		1.00	
25–34	1198	1.08 (0.70–1.67)	0.719	0.99 (0.64–1.53)	0.961
35–44	1093	1.31 (0.85–2.01)	0.223	1.07 (0.69–1.66)	0.760
≥45	453	1.87 (1.19–2.92)	0.006	1.36 (0.86–2.15)	0.186
**Gender (Third gender excluded)**					
Male	1708	2.15 (1.77–2.61)	<0.001	2.08 (1.69–2.57)	<0.001
Female	1198	1.00		1.00	
**Ethnicity**					
Upper caste groups	1268	1.00		1.00	
Relatively advantaged *janajati*	453	0.73 (0.56–0.96)	0.022	0.94 (0.71–1.25)	0.684
Disadvantaged *janajati*	596	0.92 (0.73–1.16)	0.485	1.05 (0.83–1.33)	0.672
*Dalit*	435	0.96 (0.75–1.23)	0.746	1.02 (0.79–1.32)	0.878
Disadvantaged non *dalit* terai caste groups	105	0.94 (0.58–1.52)	0.816	1.07 (0.66–1.73)	0.798
Religious minorities	49	1.38 (0.78–2.47)	0.271	1.04 (0.55–1.93)	0.912
**Usual place of residence**					
Same as ART district	1284	1.00		1.00	
Other than ART district	1622	1.42 (1.19–1.70)	<0.001	1.45 (1.19–1.76)	<0.001
**Partner's HIV status**					
+ ve	644	1.00		1.00	
- ve	366	1.19 (0.87–1.62)	0.265	1.06 (0.77–1.45)	0.710
Unknown	1896	1.22 (0.98–1.52)	0.079	1.15 (0.92–1.44)	0.217
**WHO clinical stage**					
Stage I	460	1.00		1.00	
Stage II	881	1.08 (0.71–1.64)	0.723	0.79 (0.51–1.21)	0.271
Stage III	1154	3.54 (2.45–5.11)	<0.001	1.67 (1.13–2.46)	0.010
Stage IV	411	6.83 (4.67–9.99)	<0.001	2.21 (1.45–3.36)	<0.001
**Performance scale**					
A (Normal)	1769	1.00		1.00	
B (Bedridden <50%)	814	2.57 (2.09–3.16)	<0.001	1.92 (1.52–2.41)	<0.001
C (Bedridden >50%)	323	6.90 (5.57–8.55)	<0.001	3.82 (2.95–4.94)	<0.001
**Bodyweight**^**b**^ **(kg)**	2906	1.05 (1.04–1.06)	<0.001	1.04 (1.03–1.05)	<0.001
**CD4 cell count at the start of ART** **(cells/mm**^**3**^**)**					
< 150	1526	3.12 (1.55–6.30)	0.001	2.14 (1.05–4.34)	0.036
150–199	485	1.17 (0.56–2.46)	0.677	1.22 (0.58–2.58)	0.605
200–349	809	0.75 (0.36–1.59)	0.459	0.98 (0.46–2.07)	0.958
≥350	86	1.00		1.00	
**Treatment switched to 2**^**nd**^ **line regimen**					
Yes	74	1.00		1.00	
No	2832	2.85 (1.27–6.38)	<0.001	3.05 (1.35–6.90)	0.008
**TB treatment during ART**					
No	2424	1.00	1.00		
Yes	482	1.78 (1.46–2.17)	<0.001	1.07 (0.87–1.31)	0.529

^a^BPKIHS: BP Koirala Institute of Health Sciences; STIDH: Sukra Raj Tropical and Infections Disease Hospital; WRH: Western Regional Hospital; BZH: Bheri Zonal Hospital; SZH: Seti Zonal Hospital

^b^Continuous variable

Compared with those receiving ART from SZH, Kailali, PLHIV receiving ART from BPKIHS, Dharan (adjusted hazard ratio [AHR] = 0.33, 95% CI: 0.19–0.56), STIDH Kathmandu (AHR = 0.60, 95% CI: 0.44–0.82) and WRH, Kaski (AHR = 0.71, 95% CI: 0.51–0.97) were less likely to die. Males were two times more likely to die than females (AHR = 2.08, 95% CI: 1.69–2.57). Those who came for treatment from districts other than the one where ART center is located were more likely to die than those residing in the same district (AHR = 1.45, 95% CI: 1.19–1.76). Compared with those initiating ART at WHO clinical stage I, PLHIV initiating treatment at stage III (AHR = 1.67, 95% CI: 1.13–2.46) and stage IV (AHR = 2.21, 95% CI = 1.45–3.36) were more likely to die. Compared to those with baseline performance scale A at the start of ART, PLHIV with performance scale B (AHR = 1.92, 95% CI: 1.52–2.41) and C (AHR = 3.82, 95%CI: 2.95–4.94) had higher chances of death. For each kilogram decrease in baseline bodyweight, the risk of mortality increased by 4% (AHR = 1.04, 95% CI: 1.03–1.05). PLHIV with CD4 cell count less than 150 cells/mm^3^ were around two times more likely to die than those with CD4 cell count ≥350 cells/mm^3^ at the start of ART (AHR = 2.14, CI: 1.05–4.34). Likewise, the risk of death was three times higher among those who were not switched to second line regimen compared with those who were receiving first line drugs (AHR = 3.05, 95% CI: 1.35–6.90).

## Discussion

Since the start of ART services in 2004, Nepal has continuously expanded the number of ART centers, all of which serve an estimated 14,544 PLHIV (currently on ART as of July 2017) [19]. This retrospective cohort study analyzed 10 years of data of PLHIV on ART from five large ART centers with the aim to help understand mortality and its predictors.

Nearly 20% of those enrolled on ART had died within the 10-year period. ART center location was a significant predictor of mortality with risk of death higher in clients of ART centers from the Mid and Far-Western region. There were significant differences in median CD4 cell count and WHO clinical staging at baseline between male and female clients attending these ART centers. Males had lower CD4 cell count and advanced disease stage (WHO clinical stage IV) at baseline as compared to females (S1 Table), indicating late diagnosis and treatment. Clients attending BZH, Banke, and SZH, Kailali near to the Indian border are poor and mostly migrant workers to India with no routine access to ART services. Anecdotes from PLHIV depict situations where Nepali migrants working in India were too sick to work and were sent home. Upon return, they are more likely to be enrolled in ART sites closer to the border. Over time, their wives also become infected. While the men die from advanced infection, their wives get better due to relatively early enrollment in ART. This might explain higher mortality among men in this region. A similar study [23] from Far-Western Nepal reported 11.7% deaths, lower than what was observed in this study at SZH, Kailali (21.4%). This lower proportion may be attributed to the inclusion of transferred out cases in that study.

The differences in performance between the ART centers could also result from differences in their clinical practice. Albeit guided by the national guidelines, actual clinical practice might differ in these centers due to several factors. The client load was highest in STIDH followed by WRH, SZH, BPKIHS and BZH. The greater flow of clients in certain centers compared to others might influence service quality and hence the risk of mortality but it cannot be concluded without further investigation. Over the years, the varying presence of Community Care Centers (CCC), Community Home Based Care services and support from non-governmental organizations to these ART centers in drug dispensing and provision of transportation incentives to PLHIV on ART might also explain the differences. This association, however, cannot be categorically confirmed as it was not assessed by the study and can be recommended for future studies to explore.

Similar to this study, mortality rate of 6.3 per 100 person-years over a five year-study period was reported in Far-Western Nepal [23]. Trend of mortality by follow-up time intervals was also similar to previous findings [23, 24, 43, 44]. The overall mortality rate was higher than that for high-income countries such as Switzerland [45] but it was comparable to lower middle income countries such as India [43] with similar resource constraints. Compared to high income countries, Nepal has a weaker health system, poorly trained health personnel, higher barriers to health care coverage, lower ART program coverage and people have poorer health seeking behavior, in general, which may explain the higher mortality.

Mortality rate was highest in the first three months of follow-up since ART initiation and decreased with increase in follow-up time intervals. High early mortality has been reported previously in other resource poor settings such as Northwest Ethiopia [22], Tanzania [24], Sub-Saharan Africa [25] and Far-Western Nepal [23]. This could be a result of late presentation (e.g. lower CD4 cell count) and late initiation of ART, comorbid conditions at ART initiation, poor adherence, stopping to take drugs particularly among those with less readiness to initiate ART, opportunistic infections, nutrition, and limited diagnostic and treatment facilities. In addition, poverty could also be one of the underlying factors contributing to poor access and consequently late presentation.

Globally, different causes of mortality among PLHIV have been documented. Wasting syndrome, tuberculosis, acute bacterial infections, malignancies, anemia, cerebral toxoplasmosis and immune reconstitution disease were the major AIDS related causes of death [25, 46]. While cardiovascular disease, liver disease and non-AIDS defining cancer were the non-AIDS related causes [47, 48]. This study reported all-cause mortality. Assigning causes of death retrospectively was not possible because clinical history of patients at death were not documented in registers. An approach to documenting cause of death can be useful. Studies using verbal autopsies might generate further insight and could also be useful in identifying HIV care continuum gaps [49].

Growing body of evidence on gender and ART shows more women accessing ART than men [21, 50, 51] and higher mortality among men than women [50, 52–54]. In this study, the male mortality rate was almost twice that of female. Similar results were reported by other studies [23, 24, 55, 56]. In Nepal, most of the migrant workers as well as injecting drug users are male with high risk of HIV acquisition resulting from their unsafe behavior. This is particularly true for the period of this study. Deaths in these groups might have contributed to higher mortality among men. Additionally, these groups may be economically challenged which adversely affects their nutrition status, all contributing to higher mortality.

However, a study in Northwest Ethiopia reported better survival of male than female [22] and in contrast to more women accessing ART than men, more men than women were accessing ART services in that study. Assumptions on the mechanisms of association have been made in the past, however, systematic investigation is needed to better understand gender differences to improve health outcomes of PLHIV.

For more than half of the PLHIV on treatment, the usual place of residence was not the same district as the ART center which might mean that they had to travel longer distances to access ART which is an added burden. This may have consequently, led to poor adherence, retention and follow-up clinical visits including lab tests. This may have been more common among MLM who, for very long, had to access ART services from districts other than where they reside and possibly among FSW, who are a very mobile population. Migrants might have also constantly returned to India after ART initiation, only returning to their area of residence on a seasonal basis. Routine access to ART is critical for survival. Remote residence, difficult topography and lack of transportation hinder timely access to services.

Performance scale, body weight, WHO clinical stage and CD4 count at the start of ART are critical clinical characteristics for survival and were significantly associated with mortality. Similar findings on CD4 count and WHO clinical staging were observed in other studies as well [24, 44, 55, 57, 58]. Relatively large proportions of PLHIV were in poor clinical condition at ART initiation. Around 57% were on WHO stage III and IV, thus were at an increased risk of dying [59, 60]. Likewise, 44.3% had performance scale B or C and 70.6% had CD4 cell count less than 200 cells/mm^3^. With worsening WHO clinical staging and decreasing CD4 cell count, immune system is compromised resulting in higher chances of becoming infected with opportunistic infections. A lower body weight could be a result of all these factors including poor nutrition. Early diagnosis and routine monitoring of viremia and CD4 counts and scale up of free treatment provision in low income settings could help reduce mortality [21].

Risk of mortality was three times higher among those not switching to second line regimen. Access to second line regimen and timely treatment switch with better regimen matching should improve health. For ART programs in low income countries, viral load testing is not available for routine monitoring of clients. In Nepal, viral load testing services are available in three sites, of which only two were operational as of September 2018. In its absence, the identification of treatment failure is dependent on clinical and immunological criteria. The longer the person is on failing regimen the higher the mortality risk [61]. Routine viral load testing for clients on first-line regimen has shown to be effective in identifying treatment failure at earlier stages leading to less delay in switch to second-line regimens [62]. Early Warning Indicators are also equally useful in monitoring of HIV drug resistance which is critical for regimen switching. These are quality of care indicators which specifically assess factors at individual ART clinics associated with emergence of HIV drug resistance [63].

Some limitations should be considered when interpreting the findings of this study. This study collected retrospective data recorded for regular ART program monitoring purposes. Data on all potentially important predictors of mortality were not available which restricted data only to those available in the registers, a large number of forms were incomplete and information was not recorded for many variables. People not included in the study for lack of completeness of registers may have differed from those who were included. Second, although the largest ART centers with respect to the number of PLHIV on ART were sampled, the results may or may not be generalizable to the rest of the population served by other centers. These centers may differ in terms of access and quality of services available and those who visit other centers may differ in background characteristics. Third, a large proportion of clients were lost to follow up with unknown mortality status. This might have precluded an accurate estimation of mortality. Fourth, CD4 count eligibility criteria for ART initiation has been revised overtime. Initially PLHIV were enrolled based on CD4<200, then CD4<350 and CD4<500 cells/mm^3^ and now everyone is treated. This has not been considered during the analysis. Fifth, this study covered the early period of ART roll-out in Nepal, during which the country faced its own systemic challenges such as lack of trained human resource, limited provision of widespread supporting diagnostic tools including CD4 and viral load tests, early adopters being mostly late initiators, general hesitancy among the people to initiate ART fearing lifelong dependency, constantly shifting ART guidelines including higher CD4 thresholds, limited availability of supportive care system in the community, stigma resulting in late initiation, other treatment challenges and slow decentralization of ART sites. These factors should be taken in to account when interpreting the results of this study.

To our knowledge this is the first large scale study conducted in several ART centers across Nepal that measured mortality and associated factors in PLHIV on ART. Data since the start of ART services in Nepal have been collected and analyzed, findings should reflect the complete picture. This study has several implications for the improvement of ART program in Nepal as well as for other programs that operate in low resource settings. First, the expansion of ART centers should continue to increase access and to enroll PLHIV on ART before their clinical characteristics deteriorate. With test and treat guidelines in place, the country should aim for further decentralization of the sites so that lower level health facilities, at least in the high epidemic areas provide ART services.

Second, to improve outcomes at first three months of ART initiation, case managers should be mobilized, backed by CCC that provide care to PLHIV to help in determining and ensuring ART readiness among the PLHIV and to help them adapt to their regimen in the initial months of ART through regular follow up and referral to care. Such mechanism could be expanded in all districts where ART centers are located. Case managers can provide personalized services, facilitate linkage with treatment sites, encourage early initiation especially in the case of key populations, support to make early decision to initiate treatment, follow-up for adherence, and ensure timely viral load monitoring. They can also provide the much needed treatment literacy. In addition, the more crowded health facilities along with case managers can provide differentiated care to the PLHIV on treatment. This will ensure a more tailored and personalized approach to each PLHIV. Other interventions such as improved nutritional support and socio-economic support may also be needed. Linkages between ART, case managers and CCC should be further strengthened.

Third, a unique identification system can track PLHIV throughout the treatment cascade. This should prevent duplication, facilitate easy tracking and help minimize lost to follow up cases. Fourth, recording and reporting practices at the ART centers need to be improved. In the absence of complete forms and datasets, systematic evaluation cannot be performed. Database should be periodically assessed for missing information. Moreover, an electronic central data hub system may be designed which will be useful in tracking loss to follow-up and transfer out cases. ART centers need to be have appropriate and adequately trained human resource.

## Conclusions

Following ART initiation in adult PLHIV, mortality rate was high, particularly in males and gradually decreased over time. Poor baseline clinical characteristics (i.e. WHO clinical stage, performance scale, bodyweight and CD4 count) were significantly associated with higher mortality. Additionally, those receiving ART from centers in Far-Western part of Nepal, male and residing in district other than the one where ART center is located had higher risk of death. Switching treatment to second line regimen reduced the risk of death. Important predictors of mortality must be addressed to improve outcomes of long term ART. Increase in ART coverage with decentralization of sites to lower levels including community dispensing, differentiated and improved service delivery, and initiation of ART at a less advanced disease stage may reduce early mortality.

## Supporting information

S1 Table Differences in baseline CD4 cell count and WHO clinical stage IV between male and female PLHIV attending ART sites in the western part of Nepal. (DOCX)
