# Supplementary material for: Predictors of mortality in adult people living with HIV on antiretroviral therapy in Nepal: A retrospective cohort study, 2004-2013
Source: PLoS One. 2019 Apr 23;14(4):e0215776. doi: 10.1371/journal.pone.0215776 (PMC6481250; doi:10.1371/journal.pone.0215776)
Supplement: S1 Table — (DOCX) [file pone.0215776.s001.docx]

**S1 Table: Differences in baseline CD4 cell count and WHO clinical stage IV between male and female PLHIV attending ART sites in the western part of Nepal**

| **Characteristics** | **Male** | **Female** | **P-value^*^** |
| --- | --- | --- | --- |
| **CD4 count (cells/mm^3^)** |  |  |  |
| Seti Zonal Hospital *(Median)* | 134 | 169 | <0.001 |
| Bheri Zonal Hospital *(Median)* | 129 | 177 | 0.028 |
| **WHO clinical stage IV** |  |  |  |
| Seti Zonal Hospital *n (%)* | 66 (86.8) | 10 (13.2) | <0.001 |
| Bheri Zonal Hospital *n (%)* | 24 (85.7) | 4 (14.3) | <0.001 |
| *^*^Median test for CD4 cell count and fisher’s exact test for WHO clinical staging* | | | |
